# Tackling Kinesiophobia in Chronic Shoulder Pain: A Case Report on the Combined Effect of Pain Education and Whole-Body Cryostimulation

**DOI:** 10.3390/jcm13072094

**Published:** 2024-04-03

**Authors:** Angelo Alito, Mariachiara Elisabetta Cifalinò, Jacopo Maria Fontana, Federica Verme, Paolo Piterà, Paolo Capodaglio

**Affiliations:** 1 Department of Biomedical, Dental Sciences and Morphological and Functional Images, University of Messina, 98125 Messina, Italy; alitoa@unime.it; 2 Research Laboratory in Biomechanics, Rehabilitation and Ergonomics, IRCCS, Istituto Auxologico Italiano, San Giuseppe Hospital, Piancavallo, 28824 Verbania, Italy; mchiara.cif@gmail.com (M.E.C.); f.verme@auxologico.it (F.V.); p.capodaglio@auxologico.it (P.C.); 3 Department of Clinical and Biological Sciences, University of Turin, Orbassano, 10043 Torino, Italy; p.pitera@auxologico.it; 4 Department of Surgical Sciences, Physical and Rehabilitation Medicine, University of Torino, 10121 Torino, Italy

**Keywords:** chronic shoulder pain, kinesiophobia, pain education, rehabilitation, whole-body cryostimulation

## Abstract

Background: Chronic shoulder pain may cause significant functional disability and reduced psychosocial well-being. Detailed Case Description: In this case, we propose the use of pain neuroscience education and whole-body cryostimulation (WBC) to treat a 64-year-old woman with severe functional limitations and chronic right shoulder pain. The aim was to overcome kinesiophobia and improve her motor function, autonomy, and quality of life. Functional and clinical assessments were conducted at admission, discharge, and at a one-month follow-up via phone call. The patient’s global health, shoulder function, and quality of life showed improvement during hospitalisation and were maintained after one month. Discussion: Pain education is crucial in managing chronic shoulder pain, especially in addressing kinesiophobia and promoting positive patient outcomes. In this context, WBC was used as a supplementary treatment to traditional pain relief and exercise tolerance therapies. This can help individuals to participate more actively in their rehabilitation process, ultimately promoting functional recovery and an improved quality of life. Conclusion: The combination of cryostimulation, tailored physical exercises, pain education, manual therapy, and psychological support created a synergistic effect that addressed both the physical and psychological aspects of pain and kinesiophobia.

## 1. Introduction

Chronic pain is recognised as a condition lasting beyond the physiological healing period persisting or recurring for more than 3–6 months [1,2].

Shoulder pain is a common musculoskeletal condition accounting for approximately 1.3% of all general practice presentations [3,4]. Incidence and prevalence rates tend to increase with age (degenerative processes), in women (anatomical, biomechanical and hormonal factors), in lower socioeconomic groups (reduced access to healthcare structures, physically demanding occupations), and in psychologically stressed populations (somatisation and increased pain sensitivity) [5,6].

Identifying the cause of a shoulder pain can be difficult due to the large number of structures such as bones, ligaments, tendons, and bursae that can be involved in the onset of pain or dysfunction [7]. In addition, these parts must maintain bone congruence, which guarantees dynamic stability for all the range of motion (ROM) [8].

Patients with chronic shoulder pain often experience significant functional disability and reduced psychosocial well-being, and to best manage the shoulder condition, accurate diagnosis is essential, and clinical assessment and imaging studies must be carefully considered [9].

Many shoulder pathologies such as rotator cuff, long head biceps tendon or labrum lesions, bursitis, adhesive capsulitis, osteoarthritis, acromioclavicular joint disease, or instability can present with similar symptoms (e.g., pain, weakness, ROM limitation) [10]. As a result, there is a wide range of treatment options, from conservative management, including activity modification, systemic (e.g., analgesics or anti-inflammatories) or local (e.g., intraarticular injections) medications, to manual treatment, therapeutic exercise, and surgical treatment [8,11].

Indeed, many biopsychosocial factors have been linked to the chronicisation of pain, leading to anxiety, poorer recovery and quality of life (QoL), and sometimes to pain avoidance beliefs [12,13].

Kinesiophobia is an excessive, irrational, and disabling fear of movement or activity due to a sense of vulnerability to painful injury or re-injury and is often associated with avoidance behaviours such as altered motor patterns that may limit actual functional capacity [14,15].

To prevent physical damage, people learn to avoid movements and activities that they find painful and to adopt different or alternative behaviours [16,17].

This can lead to reduced shoulder range of motion and stiffness, potentially leading to further limitation of ROM and functionality, resulting in muscle weakness and disuse, deconditioning of normal movement and adoption of maladaptive motor patterns [14]. In addition, the maintenance of these symptoms can lead to psychological distress, which can exacerbate the perception of pain, perpetuating this vicious cycle, prolonging rehabilitation time, and limiting participation in therapeutic exercise and recovery [18].

Higher levels of fear-avoidance beliefs are associated with greater levels of motor impairment and disability, so communication between healthcare providers and patients, including education, disease information, treatment goals and reassurance, may also help recovery and play an important role in rehabilitation outcomes [19,20,21].

In this context, pain neuroscience education (PNE), as an ongoing process based on an understanding of pain physiology, sensitisation, and its role in chronic pain, can represent a core strategy in pain management, aiming to reconceptualise the understanding of pain as less threatening [22,23,24].

Several studies have shown that PNE can reduce pain, disability, catastrophising and kinesiophobia in the short to medium term [25,26] and there are different types of treatment modalities, such as individual education through oral dialogue, group treatments, and information through the provision of a booklet and/or printed materials [27].

The literature has shown that the most effective intervention is the individual interview, which allows treatment to be specific and individualised for each patient, creating a patient–clinician therapeutic alliance that improves treatment outcomes [28].

Furthermore, the multifaceted interactions and consequences of chronic pain and kinesiophobia require a multidisciplinary approach that addresses the complex interplay between physical, psychological, and social factors [14,29].

Because of its rapid effect on overall well-being by modulating pain and improving mobility, whole-body cryostimulation (WBC) has been used as an adjuvant therapy in several conditions and could play an important role in the treatment of chronic pain [30,31,32]. The effects of cryostimulation have been shown to be beneficial in several systems such as haematological, metabolic, and musculoskeletal, as well as with an anti-inflammatory effect, in post-exercise and post-traumatic recovery, pain and physical performance, and in psychological aspects [30,33]. This treatment consists of repeated daily sessions of 2–3 min in which the whole body is exposed to extremely low temperatures (−110 to −140 °C) in cryogenic chambers with the aim of reducing inflammation and vasoconstriction, modulating pain transmission, and regulating the release of neurotransmitters [29].

The aim of this work is to provide evidence on the role of a multidisciplinary rehabilitation programme involving PNE and WBC in overcoming kinesiophobia and improving patients’ motor function, autonomy, and quality of life.

This case report followed the CARE guidelines for case reports [34].

## 2. Detailed Case Description

A 64-year-old woman was admitted to the rehabilitation department of the Istituto Auxologico Italiano, Piancavallo (VB) in December 2023 with severe functional impotence of the right shoulder. She presented with a history of diabetes mellitus type 2, cervical and lumbar discopathy, and polyarthralgia. She also reported chronic low back pain with a previous lumbar microdiscectomy about twenty years ago and left knee pain with meniscopathy and patellofemoral chondropathy.

**Figure 1:**
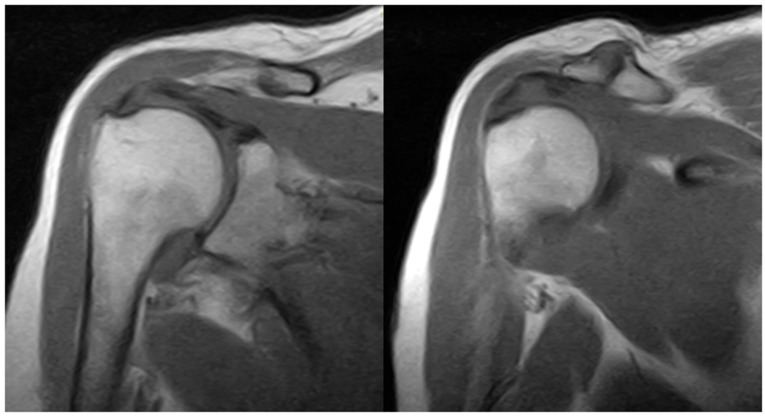
Shoulder MRI showing mild acromioclavicular arthrosis and severe supraspinatus tendinosis with intratendinous microinjuries and oedema.

The impairment of the right shoulder started in the last months of 2021 with a progressive development of pain and functional limitations. She underwent an orthopaedic consultation which prescribed restriction of shoulder movements and physiotherapy, not recommending surgery. The patient started rehabilitation treatment based on physiotherapy and mild shoulder mobilisation with no clinical improvement. In January 2022, she underwent an MRI scan due to persistent pain. This revealed mild acromioclavicular arthrosis, severe tendinosis of the supraspinatus tendon with oedema and intratendinous microinjuries in the critical area, mild tendinosis of the infraspinatus tendon with a thin film of fluid in the subacromial deltoid bursa, and a mild effusion on the tendinous sheath of the long head of the biceps (Figure 1).

Then, she underwent two cycles of physiotherapy, mainly physical therapy modalities (e.g., laser therapy, ultrasound therapy) with little clinical benefit. Meanwhile, she was taking NSAIDs to control the pain, and between June and July 2023 she underwent a cycle of three intraarticular injections of steroids with mild clinical benefit. The persistence and worsening of the pain led the patient to a progressive limitation of shoulder movements with the development of fear-avoidance behaviours until her admission to the S. Giuseppe Hospital of Piancavallo in December 2023.

At the first physiatric assessment, the patient presented with an autonomous normal gait, a swayback posture, severe antalgic limitation of active movements of the right shoulder with severe mid-range pain, reduced ROM, and significant kinesiophobia. There were no neurological symptoms (i.e., paraesthesia or sensory deficits).

At the physiotherapy assessment, the patient presented with her right arm in an antalgic position and expressed considerable concern about movement-related pain, treatment modality, and prognosis. She complained of persistent pain and increased use of her left arm to avoid painful movements and her autonomy with activities of daily living (ADLs) was very limited, often requiring assistance. Shoulder examination revealed severe antalgic ROM limitation, particularly in flexion, abduction, and internal rotation (NRS 10/10), combined with a fear of movement.

The patient underwent multidisciplinary treatment, including a dietary plan, physiotherapy sessions, and psychological support and, in addition, given the repeated failures of the previous approach about pain management, and given the absence of contraindications, the physicians suggested the implementation of WBC treatment.

The physiotherapy sessions focused on therapeutic exercise, manual therapy, and pain education, with the aim of restoring full range of motion and desensitising chronic pain. The rehabilitation program started with passive shoulder mobilisation in a pain-free ROM, progressed to an active/assisted modality with acceptable pain, then progressive isotonic exercises to gradually return to physiological movements. Finally, a progressive strength training program was implemented based on the patient’s positive feedback.

Treatment sessions included individualised pain education through verbal interviews discussing the physiology of the pain system, the characteristics, purpose, and origin of acute and chronic pain, the mechanisms of pain chronicisation and the potential factors contributing to central sensitisation such as emotions, stress, illness, perception, pain cognition, and pain behaviour.

In addition, the patient performed five daily WBC sessions (2 min/−110 °C) in a cryo-chamber (Artic, CryoScience, Rome, Italy) with an optimal treatment adherence, subjective reduction in pain and satisfaction, and no adverse effects reported.

Functional and clinical assessments were performed at admission and discharge. Global function was assessed using the 6 min walk test (6MWT) [35], overall strength using the hand grip test (HGT) [36], and general well-being using the Psychological General Well-being Index (PGWBI) [37]. Shoulder evaluation was performed by measuring ROM with a goniometer, the Disabilities of the Arm, Shoulder and Hand (DASH) questionnaire [38], and the Shoulder Pain and Disability Index (SPADI) [39]. In addition, the Short-Form Health Survey 36 (SF-36) [40] was used to measure health-related quality of life (HR-QoL) and the Tampa Scale for Kinesiophobia (TSK) [41] to quantify fear of painful movement. During the hospital stay, the patient reported a subjective improvement in physical performance, shoulder movements, and a global reduction in pain. A global view of the assessments is provided in Table 1.

**Figure 2:**
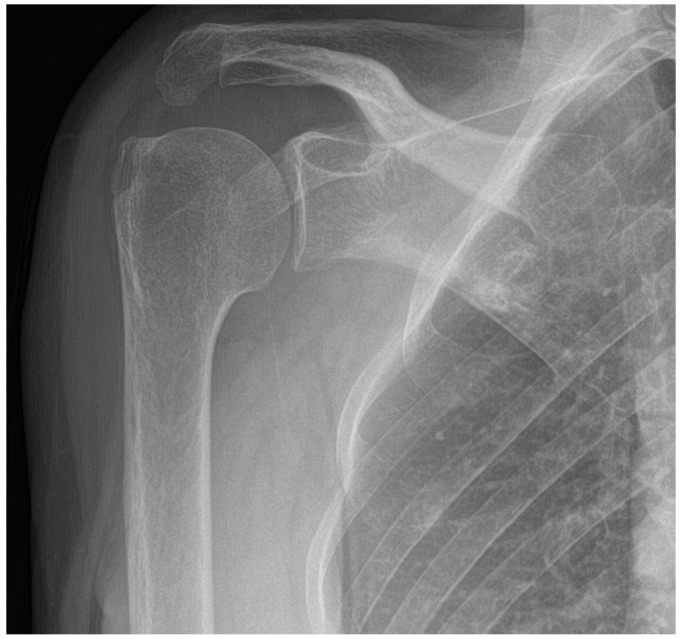
Radiograph of the right shoulder in extrarotation showing mild acromioclavicular arthrosis.

**Figure 3:**
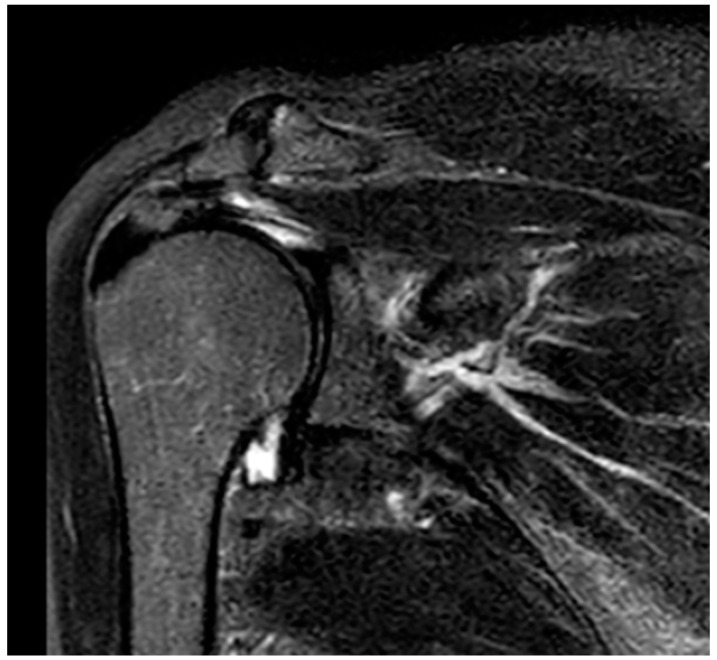
The pre-discharge MRI shows an osteofibrous sleeve of the acromion-clavicular joint with impingement of the supraspinatus tendon with a full-thickness anterosuperior lesion.

The patient also underwent an X-ray exam of the right shoulder that showed no focal bone morpho-structural changes and mild evidence of acromioclavicular arthrosis (Figure 2) and an additional MRI scan that showed an osteo-fibrous sleeve of the acromion-clavicular joint with impingement of the tendon of the supraspinatus muscle with a full-thickness anterosuperior lesion. In addition, tendinosis of the tendon of the subscapularis muscle with bursitis was noted (Figure 3).

**Figure 4:**
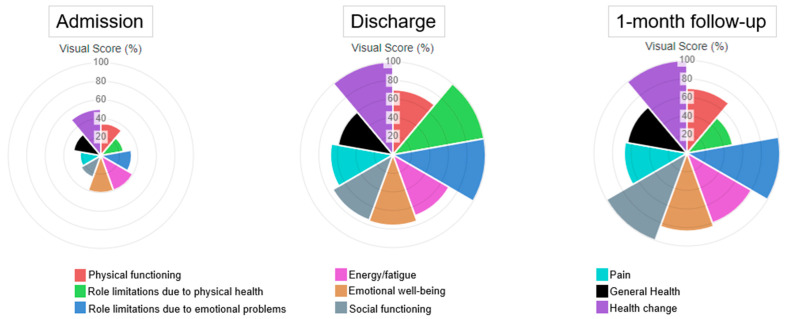
Visualising well-being: SF-36 graphical representation capturing dynamic changes in quality of life at admission, discharge and one-month follow-up; https://orthopowertools.com/SF36 (accessed on 26 January 2024).

**Figure 5:**
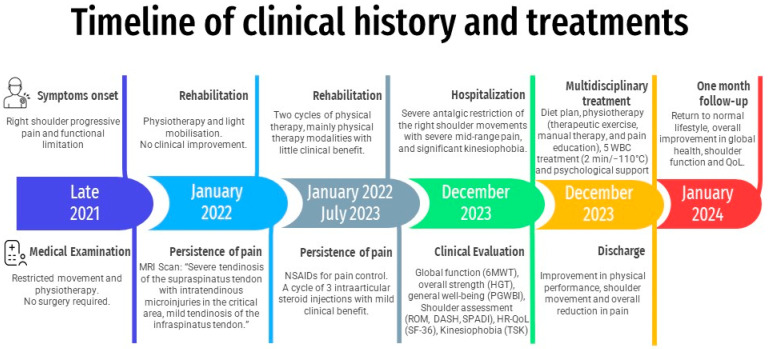
Reporting medical progress: A timeline of patient clinical history and interventions.

After discharge, a follow-up telephone call was made to inquire about general health, maintenance of results achieved during hospitalisation, and to re-complete the DASH, SPADI, TSK and SF-36. The patient reported that she returned to her normal lifestyle with an overall improvement in global health, shoulder function (Table 2), and quality of life (Table 3, Figure 4). A comprehensive timeline of clinical history and interventions is shown in Figure 5.

## 3. Discussion

Chronic pain is a disabling condition that affects millions of people worldwide, not only physically but also emotionally and psychologically [1,42]. As well as physical discomfort, it can lead to fear, anxiety, and avoidance behaviours [43]. As a result, kinesiophobia is a common problem among people experiencing chronic pain, with a significant impact on quality of life, leading to reduced mobility, muscle weakness, and even further pain and disability [44].

Understanding kinesiophobia is an important component of chronic pain management, as it can greatly affect a patient’s motivation to comply with physical therapy and rehabilitation programs [18,45].

Healthcare providers can assist patients in overcoming their fears and gradually increasing their confidence in movement and exercise by addressing and educating them [46,47].

In this case study, the patient suffered from chronic shoulder pain for a few years, and the initial moderate pain and limitation of movement resulted in the patient consulting several medical specialists. The failure of repeated treatments and maintenance of pain led to a progressive reduction in shoulder use and function as well as mood.

Over time, the chronicisation of pain evolved into a significant level of kinesiophobia, with fear and hesitation to move the right shoulder or engage in any form of exercise, exacerbated by previous experiences of pain during physical activity.

The physiotherapist used a personalised approach, explaining the underlying mechanisms of pain and the importance of gradually challenging her fear of movement, addressing the patient’s beliefs and gradually changing her perception of movement from a source of fear to a pathway to recovery.

To further address the patient’s chronic shoulder limitation and pain, a WBC cycle was implemented in this multidisciplinary approach with the aim to facilitate the rehabilitation process.

Over time, this rehabilitation approach resulted in a quite complete resolution of symptoms and improvement in the patient’s confidence in physical activity, regaining shoulder mobility and function even one month after discharge, with the patient maintaining most of the achieved improvements.

Interestingly, the psychological effects of using an innovative device may play a role in better adherence to rehabilitation treatment and the known effects of WBC on pain, especially in the short term, may have an adjunctive effect to physical therapy.

WBC has gained attention for its potential benefits in the management of chronic pain by inducing a systemic response, leading to various physiological and biochemical changes that can effectively modulate pain perception, reduce inflammatory markers, and improve overall pain tolerance [30,32]. It helps to reduce swelling and oedema in the affected area by inducing vasoconstriction, thus providing relief from pain and discomfort [48]. WBC may also exert local analgesic effects by reducing nerve transmission throughout the body, combined with increased endorphin concentration and norepinephrine production released from both peripheral nerve terminals and brain nuclei by sympathetic stimulation, ultimately reducing the perception of pain [49,50,51]. WBC also has significant psychosocial implications, as the release of endorphins and other neurotransmitters not only relieves pain but also plays a crucial role in reducing the psychological barriers associated with chronic pain, such as anxiety and depression [52,53].

In this context, WBC can serve as a complementary intervention to traditional pain relief and exercise tolerance therapies, enabling individuals to participate more actively in their rehabilitation process and ultimately promoting functional recovery and improved quality of life [30,54].

The results obtained in this patient are also consistent with the study by Ma and colleagues who compared physiotherapy modalities and joint mobilisation with the same treatments plus WBC in patients with shoulder adhesive capsulitis [50].

Pain education and support from healthcare professionals is essential to help people understand their active role in pain management, address common misconceptions and beliefs about kinesiophobia, reduce anxiety and develop a better attitude, and focus on the recovery process [22,55].

Studies suggest that factors such as pain catastrophising, kinesiophobia, and ineffective pain management methods play a crucial role in understanding the diversity of pain levels and physical functioning in people with chronic shoulder pain [56].

As a result, the importance of a biopsychosocial approach has been progressively emphasised, and recent approaches include therapeutic exercise, physiotherapy combined with adjuvant treatments (i.e., WBC), pharmacological pain management, and patient education [57].

Indeed, in this case study, the combination of cryostimulation with tailored physical exercises, pain education, manual therapy, and psychological support helped to create a synergistic effect that addressed both the physical and psychological aspects of pain and kinesiophobia.

However, there are some limitations to this case study, and the positive results must be interpreted with caution. Firstly, the lack of generalisability due to the nature of the study and the lack of a standardised protocol makes it difficult to establish causality or to determine the true significance of the observed results.

The patients’ positive emotional involvement in the treatment and high level of satisfaction may have played a role in the observed global functional improvement, so a placebo effect cannot be excluded. In addition, the authors cannot determine the component of the rehabilitation program that may have played the most important role in the results.

To the best of our knowledge, this is the first case reporting on the effects of a multimodal approach, involving WBC and pain education in a case of kinesiophobia in chronic shoulder pain. Future research should further investigate the long-term efficacy and optimal dosage of this combined approach, as well as its application to different patient groups and clinical settings.

## 4. Conclusions

This case report illustrates the effectiveness of a multimodal approach with the integration of physical therapy, pain education, and WBC in the management of chronic shoulder pain, particularly in overcoming both the physiological and psychosocial aspects of kinesiophobia and facilitating medium-term recovery. The combined approach of WBC and PNE is a valid strategy for the treatment of kinesiophobia, highlighting the complementary and synergistic effects of these approaches in reducing fear-avoidance behaviours and improving pain coping mechanisms. The subjective experience of the patients, from very early sessions, is that WBC provides a rapid sense of well-being and reduces the perception of pain, leading to improved compliance with rehabilitation treatments and that the combined effects of pain education and pathways play an important role in improving the patients’ health status.

## Figures and Tables

**Table 1:** Comprehensive admission and discharge assessments: A global view of functional and psychological well-being.

	Admission	Discharge
6MWT (m)	405	492
NRS (0–10)	10	3
HGT (kg)	RH 23.7 LH 19.2	RH 24.2 LH 19.6
PGWBI	50	75
Passive Right Shoulder ROM		
Flexion	120°	180°
Extension	25°	30°
Abduction	110°	180°
Internal Rotation	40°	100°
External Rotation	30°	35°

6MWT, 6 min walk test; HGT, hand grip strength test; LH, left hand; NRS, numerical rating scale; PGWBI, Psychological General Well-being Index; RH, right hand; ROM, range of motion.

**Table 2:** Measure progress over time: Admission, discharge, and 1-month follow-up scales for comprehensive assessment of patient outcomes.

	Admission	Discharge	One-Month Follow-Up
DASH (%)	79.31	28.44	25.8
SPADI (%)			
Pain	84	42	14
Disability	90	63.7	11.3
Total SPADI Score	87.7	55.4	12.3
TSK			
Activity avoidance (6–24)	19	8	7
Somatic Focus (7–28)	18	9	7
Total TSK Score (13–52)	37	17	14

DASH, Disabilities of the Arm, Shoulder, and Hand questionnaire; SPADI, Shoulder Pain and Disability Index; TSK, Tampa Scale for Kinesiophobia.

**Table 3:** Tracking quality of life outcomes: SF-36 results at admission, discharge, and 1-month follow-up, providing insights into patient well-being across the healthcare continuum.

Score (%)	Admission	Discharge	One-Month Follow-Up
Physical functioning	35	70	70
Role limitations due to physical health	25	100	50
Role limitations due to emotional problems	33.3	100	100
Energy/fatigue	40	70	80
Emotional well-being	40	76	84
Social functioning	25	75	100
Physical pain	22.5	67.5	67.5
General health	50	60	65
Health Change	50	100	100

## Data Availability

The data presented in this study are available within the article.
